# Transcriptome Analysis of the Zebrafish Model of Diamond-Blackfan Anemia from RPS19 Deficiency via p53-Dependent and -Independent Pathways

**DOI:** 10.1371/journal.pone.0071782

**Published:** 2013-08-19

**Authors:** Qiong Jia, Qian Zhang, Zhaojun Zhang, Yaqin Wang, Wanguang Zhang, Yang Zhou, Yang Wan, Tao Cheng, Xiaofan Zhu, Xiangdong Fang, Weiping Yuan, Haibo Jia

**Affiliations:** 1 Key Laboratory of Molecular Biophysics of Ministry of Education, College of Life Science and Technology, Center for Human Genome Research, Huazhong University of Science and Technology, Wuhan, Hubei, China; 2 CAS Key Laboratory of Genome Sciences, Beijing Institute of Genomics, Chinese Academy of Sciences, Beijing, China; 3 State Key Laboratory of Experimental Hematology, Institute of Hematology and Blood Disease Hospital, Chinese Academy of Medical Sciences & Peking Union Medical College, Tianjin, China; 4 Hepatic Surgery Center Tongji Hospital, Tongji Medical College, Huazhong University of Science and Technology, Wuhan, Hubei, China; UMDNJ-Robert wood Johnson Medical School, United States of America

## Abstract

Diamond-Blackfan anemia (DBA) is a rare inherited bone marrow failure syndrome that is characterized by pure red-cell aplasia and associated physical deformities. It has been proven that defects of ribosomal proteins can lead to this disease and that RPS19 is the most frequently mutated gene in DBA patients. Previous studies suggest that p53-dependent genes and pathways play important roles in RPS19-deficient embryos. However, whether there are other vital factors linked to DBA has not been fully clarified. In this study, we compared the whole genome RNA-Seq data of zebrafish embryos injected with RPS19 morpholino (RPS19 MO), RPS19 and p53 morpholino simultaneously (RPS19+p53 MO) and control morpholino (control). We found that genes enriched in the functions of hematological systems, nervous system development and skeletal and muscular disorders had significant differential expression in RPS19 MO embryos compared with controls. Co-inhibition of p53 partially alleviates the abnormalities for RPS19-deficient embryos. However, the hematopoietic genes, which were down-regulated significantly in RPS19 MO embryos, were not completely recovered by the co-inhibition of p53. Furthermore, we identified the genome-wide p53-dependent and -independent genes and pathways. These results indicate that not only p53 family members but also other factors have important impacts on RPS19-deficient embryos. The detection of potential pathogenic genes and pathways provides us a new paradigm for future research on DBA, which is a systematic and complex hereditary disease.

## Introduction

Diamond-Blackfan anemia (DBA) is a congenital anemia and broad developmental disease that develops soon after birth. The anemia results from a failure of erythropoiesis, with normal platelet and myeloid lineages, and it can be managed with steroids, blood transfusions, or stem cell transplantation [1]. DBA is the first known inherited disease that results from a defect in a structural ribosomal protein [2]. In humans, mutations in an increasing number of genes encoding RPs of the small (RPS19, RPS24, RPS17, RPS7, RPS10, RPS26) and large (RPL35A, RPL5, RPL11) ribosomal subunits have been shown to cause DBA. Moreover, RPS19 is the most frequently mutated ribosomal protein gene, which accounts for approximately 25% of DBA patients [3]. However, how the ribosomal protein mutations specifically affect hematopoiesis is still unclear.

The zebrafish (Danio rerio) is an excellent model organism for such studies because of its embryo transparency, high fecundity and fast development of organogenesis. It is notable that many of the key molecular players and events that drive organogenesis in zebrafish are evolutionarily and functionally conserved with other organisms, including mammalians [4]. Several groups have previously used zebrafish for modeling ribosomal protein knockdown in the embryo with morpholino technology or generating ribosomal protein mutants [5]. Recent studies have suggested that p53-mediated cell cycle arrest and/or apoptosis in erythroid cells could be the major factors in DBA development [3], [5], [6], [7]. For example, regarding the RPL11 mutant in zebrafish, Danilova et al propose that the unique phenotype of DBA is the sum of several abnormally regulated molecular pathways mediated by the p53 protein family and p53-independent synergistic impacts on hematological and other cellular pathways affected in DBA [6]. In RPS7-deficient zebrafish embryos, p53 was activated, and its downstream target genes and biological events were induced, including apoptosis and cell cycle arrest. Furthermore, simultaneous knockdown of the p53 protein could partially reverse the abnormal phenotype of the morphants [3]. In the RPS29 mutant, genes that are up-regulated are enriched with genes that are up-regulated by p53 after irradiation and the p53 knockdown almost completely rescues the RPS29 morphological and hematopoietic phenotypes, demonstrating that p53 mediates the effects of rps29 knockdown [5]. Knockdown of two other RP genes, RPS3A and RPL36A, result in severe morphological abnormalities with mild erythroid defects and elicited an activated p53 response. For the RPS19-deficient zebrafish, its phenotype is mediated by dysregulation of deltaNp63 and p53, and suppression of p53 and deltaNp63 alleviates the RPS19-deficient phenotypes [8]. At the same time, co-inhibition of p53 activity rescued the morphological abnormalities but did not alleviate erythroid aplasia in RPS19-deficient zebrafish. Hence, both the p53-independent and p53-dependent pathways could be responsible for the defective erythropoiesis in the zebrafish model of Diamond-Blackfan Anemia because of RPS19 deficiency [7].

In this study, we observed the hemoglobin synthesis defect at 48 hpf in RPS19 knockdown zebrafish embryos, and this defect can only be partially reversed by co-inhibition of p53 activity. To investigate the underlying regulatory mechanisms, we generated three types of zebrafish morphants by MO microinjection, including control morphants (control), RPS19 morphants (RPS19 MO), and RPS19 and p53 morphants (RPS19+p53 MO), and we performed transcriptome analysis of all of the pairs using the RNA-Seq technique. We found significant differentially expressed genes in RPS19 MO and RPS19+p53 MO compared with the controls. These genes are associated with the functions of cell cycle, hematological system and nervous system development and the skeletal and muscular disorders. Additionally, we determined the genome-wide p53-dependent and -independent genes and pathways. Our results demonstrated that members of the p53 network as well as other partners exert important impacts on RPS19-deficient embryos. The detection of potential pathogenic genes and pathways in this study will provide a new research paradigm for the study of DBA.

## Results

### Zebrafish Phenotypes of RPS19-deficient and Co-inhibition of p53 Activity using MO

**Figure 1:**
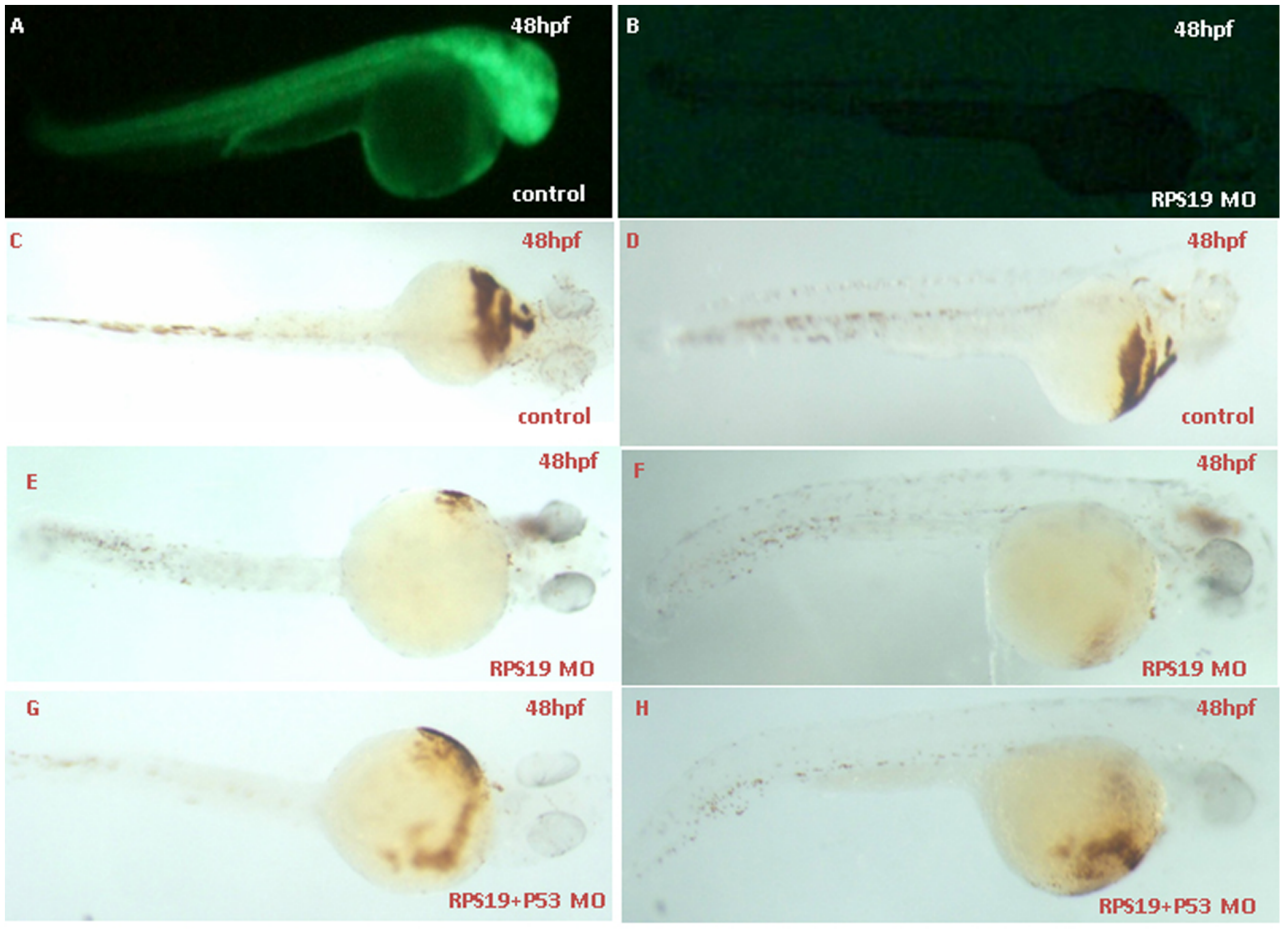
Effectiveness of RPS19 morpholinos and hemoglobin staining of embryos coinjected with Rps19 mRNA and P53 MO using o-dianisidine. The Rps19:egfp construct was made by inserting a partial sequence of Rps19 cDNA (containing 60 bps from the 5′ UTR ) with the N-terminus of egfp into modified pEGFP-N1 (the ATG codon of EGFP was removed). The sequence of RPS19 MO1 is a compliment of bp 1–24 of Rps19 cDNA. Embryos co-injected with 25 ng Rps19:egfp DNA and 5 ng control MO produced green fluorescent fusion protein (A), and expression of the fusion protein was inhibited by co-injection with 2 ng Rps19 Mo (B). O-staining results show a drastic reduction in the number of hemoglobin-stained blood cells when Rps19 is knockdown (C and D are the control, E and F are Rps19 knockdown) and partially reversed by co-injection of P53 morpholino (G and H). A, B, D, F and H are the lateral view; C, E and G are the ventral view.

Developmental defects are found in approximately 40% of DBA patients with mutations in RPS19. Zebrafish RPS19 is approximately 87.7% identical to the human homolog. As previously demonstrated, the RPS19 deficiency in zebrafish results in hematopoietic and developmental abnormalities that resemble DBA. The embryos injected with control MO did not display any morphological changes. RPS19 morphants showed an obvious ventrally bent tail and a reduction in the circulating blood cells compared with the control embryos. At 48 hpf, the hemoglobin staining results showed that hemoglobin-stained blood cells in the heart region were markedly decreased in RPS MO, which is partially rescued in RPS19+p53 MO (Fig. 1). Further *in situ* results showed that the gata1 expression level are comparable in control Mo, RPS MO and RPS19+p53 MO (Fig. S1), which is consistent with previous results [8]. In addition, we used HSCs and definitive hematopoiesis markers cmyb and runx1 to examine the expression after RPS19 morpholino (2 ng) injection or Rps19 (mo) and P53 (mo) co-injection. We did not find significant changes of the expression of cmyb and runx1 (Fig. S1), indicating the divergent functions of RPS19 and RPL22 in hematopoiesis [9]. The effectiveness of translational inhibition by RPS19 MO was confirmed by examining green fluorescent fusion protein under fluorescence microscopy (Fig. 1).

### Transcriptome Profile Analysis

**Figure 2:**
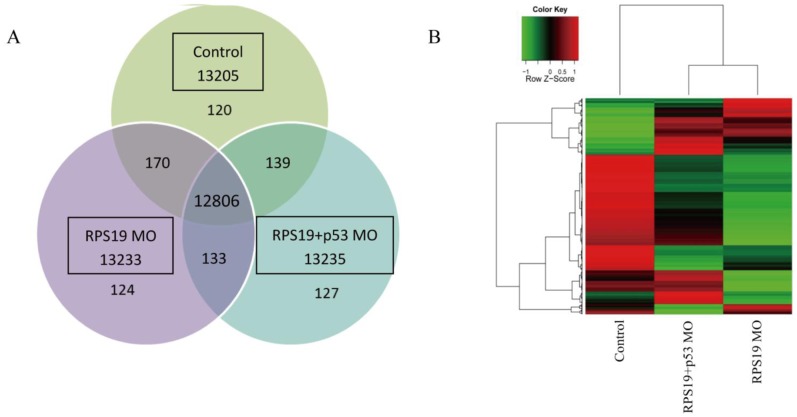
Overview of gene expression profiles of diverse zebrafish embryos. (A) Venn diagram detailing shared and distinct genes expression among three zebrafish embryo samples: control morpholino (control), RPS19 morpholino knockdown (RPS19 MO), RPS19 and p53 morpholino knockdown simultaneously (RPS19+p53 MO). (B)Unsupervised clustering of genome-wide gene expression (cutoff FPKM<1) for diverse zebrafish embryos (control morpholino (control), RPS19 morpholino knockdown (RPS19 MO), RPS19 and p53 morpholino knockdown simultaneously (RPS19+p53 MO)). Gene expression tracks use red and green to represent over- and under-expression, respectively. The pattern of the transcriptome profile of RPS19 MO is more similar to that of RPS19+p53 MO.

As previously demonstrated, the RPS19 deficiency in zebrafish results in hematopoietic and developmental abnormalities that resemble DBA. To determine the effect of RPS19 MO on the whole transcriptome and to delineate the function of p53 in RPS19-deficient embryos, we analyzed the transcript profiles using RNA-Seq. Three mRNA-Seq libraries were generated, including zebrafish embryos with control morpholino (control), RPS19 morpholino knockdown (RPS19 MO), and RPS19 and p53 morpholino knockdown simultaneously (RPS19+p53 MO). These libraries were sequenced using the Illumina Hi-seq 2000 Genome Analyzer platform with paired-end 100 base-pair tags to a depth of 35–60 million reads. We mapped the sequencing data to the latest zebrafish genome assembly version 2010 (Zv9). A total of 17–25 million reads could be mapped to the genome, representing 40%–46% of all of the generated reads (Table S1 in Tables S1). FPKM was used to estimate the transcript abundance. Overlapped and distinct gene expression among three samples was shown in Fig. 2. Unsupervised hierarchical clustering of genome-wide expression profiles (FPKM>1) was performed to examine the relationship among these three samples (Fig. 2). We observed that, compared with the control, the pattern of transcriptome profile of RPS19 MO is more similar to that of RPS19+p53 MO. The transcriptome difference between the profiles of RPS19 MO and the control is more significant than that of RPS19+p53 MO and the control. These results implied that simultaneous knockdown of p53 by co-injecting a p53 MO could partially reverse the abnormal phenotype in the RPS19 morphants.

### Transcriptome Analysis of RPS19-deficient Embryos

**Figure 3:**
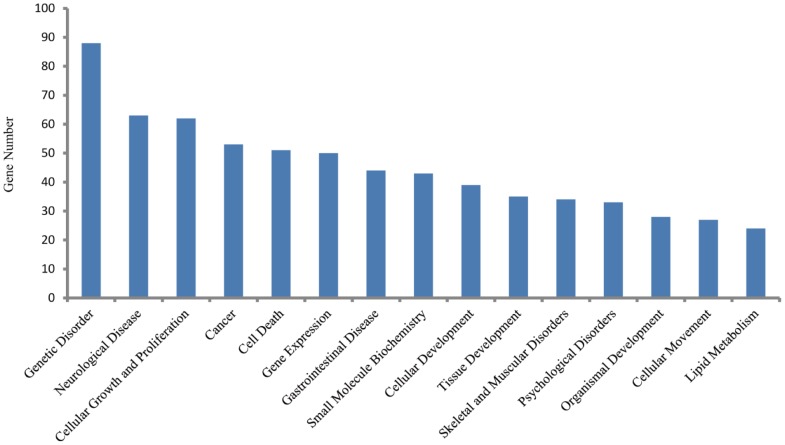
The top enriched biological functions of 359 differentially expressed genes in RPS19-deficient embryos (p-value<0.05).

**Figure 4:**
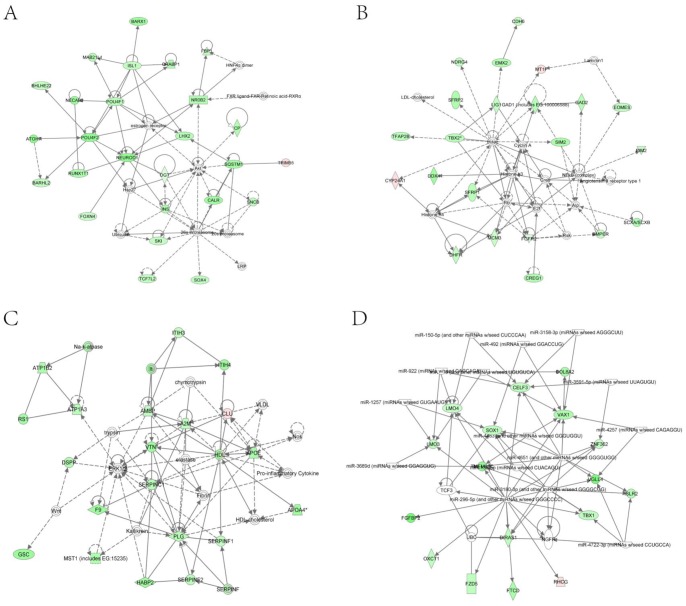
Top networks identified with IPA for the differentially expressed genes in RPS19 MO compared with the control. The pink or red nodes in the networks indicate a gene that is up-regulated in RPS19 MO, and the green color indicates genes that are down-regulated in RPS19 MO. (A) Network with the functions of embryonic development, organismal development, and cellular development. (B) Network with the functions of digestive system development and function, developmental disorder, and skeletal and muscular disorders. (C) Network with the functions of hematological system development and function, organismal functions, and cellular movement. (D) Network with the functions of nervous system development and function, tissue morphology, and cell-to-cell signaling and interaction.

To observe the transcriptome changes of RPS19-deficient embryos, we compared the differences of the whole genome RNA-Seq data between RPS19 MO and the control using the software Cufflinks. There were 47 up-regulated genes and 312 down-regulated genes in RPS19 MO compared with the control sample (fold-change>2.0, p-Value<0.05). These genes were subjected to Ingenuity Pathway Analysis (IPA) to identify the enrichment of genes in specific functional groups and pathways (IPA, http://www.ingenuity.com). The IPA accepts human UniGene IDs as one of the identifiers for data uploading and analysis. Hence, we mapped these differentially expressed genes to their human homologs using the HomoloGene database. Then, human homologs of up- and down-regulated genes of the RPS19-deficient embryos were analyzed by using IPA tools in which the gene sets are enriched for a specific function/pathway and the enrichment is represented as a ratio. The main biological functions that were enriched by differentially expressed genes are associated with genetic disorder, neurological disease, cellular growth and proliferation, cancer and cell death (p-Value<0.05). The number of enriched genes for the above GO terms is more than 50, which accounts for at least 14% of the whole set of differentially expressed genes (Fig. 3). The significantly changed genes are associated with signaling pathways, including the coagulation system, acute phase response signaling, LXR/RXR activation, intrinsic prothrombin activation pathway, and the taurine and hypotaurine metabolism (Table S2 in Tables S1). The networks constructed by differentially expressed genes are associated with cellular development, cellular function and maintenance, development disorder, hematological system development and function, and neurological disease, each of which received an enrichment score of more than 20 (Fig. 4). Since the networks with functions of ‘cellular development’, ‘cellular function and maintenance’, ‘Nervous system development and function’, and ‘cell death and survival’ are inter-connected to form a big and complex regulatory network that is controlled by a few central factors and we observed expression changes in many genes and they could not be attributed to gene mutations (the probability of multi-gene mutations appear at the same time is very low), we assume this change to be a result of abnormal expression of a few central factors that affect the central nodes in the networks. In the network analysis, we found central nodes for which the human homologs are SST, OXA1L, CLDND1, MAL, Hsp70, GCG, PLC, SOX1, VAX1, POMC, and TSH.

### Transcriptome Analysis of RPS19+p53 MO Embryos

**Figure 5:**
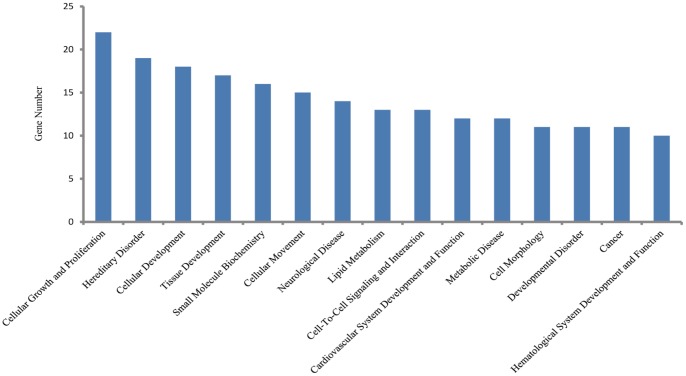
The top enriched biological functions of 147 differentially expressed genes in RPS19-deficient embryos with co-inhibition of p53 (p-value<0.05).

**Figure 6:**
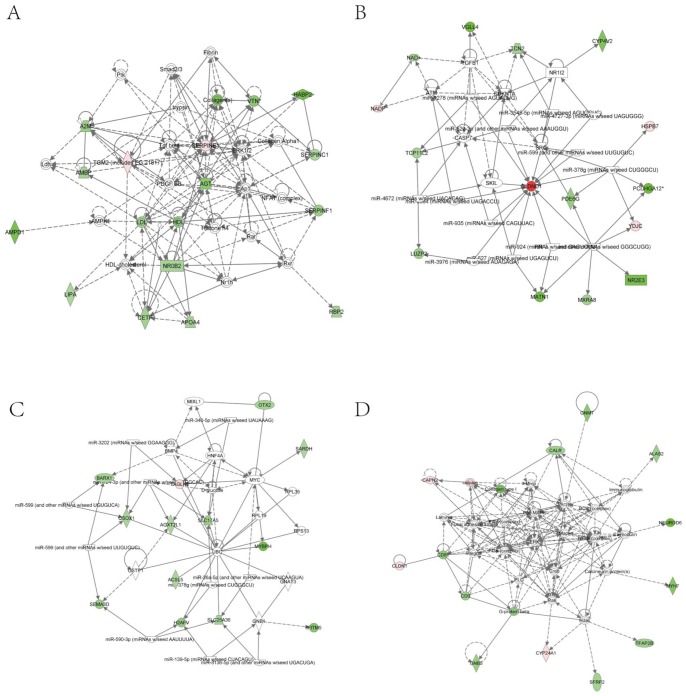
Top networks identified with IPA for the differentially expressed genes in RPS19+p53 MO compared with the control. The pink or red nodes in the networks indicate genes that are up-regulated in RPS19+p53 MO, and the green color indicates genes that are down-regulated in RPS19+p53 MO. (A) Network with the functions of lipid metabolism, molecular transport, and small molecule biochemistry. (B) Network with the functions of cell cycle, cellular development, cellular growth and proliferation. (C) Network with the functions of cancer, hematological disease, and amino acid metabolism. (D) Network with the functions of cellular development, hematological system development and function, and hematopoiesis.

To further study the role of p53 in RPS19-deficient embryos, we analyzed the RNA-Seq data of RPS19+p53 MO. Differential expression analysis is conducted by Cufflinks on the transcriptome data of RPS19+p53 MO and control/RPS19 MO. Compared with the control, RPS19+p53 MO is detected with 34 up-regulated genes and 113 down-regulated genes (fold-change>2.0, p-Value<0.05). These genes were subjected to Ingenuity Pathways Analysis (IPA) to identify the enrichment of genes in specific functional categories and canonical pathways. We found that the main biological functions of these genes are associated with cellular growth and proliferation, hereditary disorder, cellular development, tissue development and small molecule biochemistry (p-Value<0.05) (Fig. 5). The top significant canonical pathways are LXR/RXR activation, acute phase response signaling and coagulation system (Table S3 in Tables S1). The main networks constructed by these differentially expressed genes are involved in small molecule biochemistry, cell cycle, cellular development, and hematological disease. The above networks all obtained enrichment scores of more than 20 (Fig. 6). In addition, the networks with functions of ‘hematological disease’, ‘small molecule biochemistry’, ‘molecular transport’, and ‘cellular development’ are found to be connected. We also found several central nodes in these networks, the human homologs of which are TGM2, A2M, LDL, HDL, Collagen, AGT, SERPINF1, NROB2, CETP, HBZ, hemoglobin, CLDND1, calpain, CD81, and G-protein beta.

However, by comparing the transcriptome data of RPS19+p53 MO and RPS19 MO, we found only 17 up-regulated genes and 11 down-regulated genes that were significantly expressed in RPS19+p53 MO embryos (fold-change>2.0, p-Value<0.05), which is much less than the number of significant differentially expressed genes of RPS19 versus the control. These results demonstrate that co-inhibition of p53 cannot rescue the abnormalities completely in RPS19-deficient embryos. The main enriched biological functions of these genes, by identified IPA, were associated with cancer, cell death, cellular growth and proliferation, cell signaling and small molecule biochemistry. Moreover, the canonical pathways are enriched on methane metabolism, DNA double-strand break repair by homologous recombination, stilbene, coumarine and lignin biosynthesis (data not shown). These functions might be corrected by p53 MO for RPS19-deficient embryos, which are significantly influenced by the expression of p53.

### Changed Hematopoietic Genes in RPS19 MO and RPS19+p53 MO Embryos

To observe the defects of the hematopoietic functions in RPS19-deficiency embryos, we detected the differentially expressed hematopoietic genes by searching the keyword ‘hematopoietic’ while using the software AmiGO in the Gene Ontology database (http://www.geneontology.org/). We observed 12 GO terms that are associated with ‘hematopoietic’ and 177 genes in the latest annotated zebrafish genome. We matched our data to these genes, which have the GO term keyword ‘hematopoietic’. In the RPS19-deficient embryos, we found that no hematopoietic gene was up-regulated significantly, but 5 hematopoietic genes were down-regulated highly and significantly, such as bmper, fzd5, nrp1a, sema3d, and tbx1. The specific bio functions of these 5 genes are ‘hematopoiesis’(bmper), ‘T cell differentiation in thymus’(fzd5), and ‘thymus development’ (nrp1a, sema3d, tbx1). To determine whether co-inhibition of p53 would alleviate hematopoietic defect, we also matched the differential expressed genes in RPS19+p53 MO versus RPS19 MO to the 177 genes associated with hematopoietic in The Gene Ontology database. Interestingly, there was only one hematopoietic gene, fzd5, which was down-regulated in RPS19 MO, but was significantly up-regulated in RPS19+p53 MO (Table S4 in Tables S1). Previous studies showed that in zebrafish, fzd5 was associated with not only eye and retina development, but also canonical Wnt signaling, T cell differentiation in thymus and early liver formation [10], [11] and thus participated in hematopoiesis [12], [13]. As the expression of fzd5 is reversed by co-inhibition of p53, hematopoiesis and other functions of fzd5 are reversed by p53 MO. In combination with our hemoglobin staining results of RPS19+p53 MO, we concluded that the hematopoietic defects could not be remedied completely through co-inhibition of p53 in RPS19-deficient embryos.

### p53-dependent and -independent Genes and Pathways

**Figure 7:**
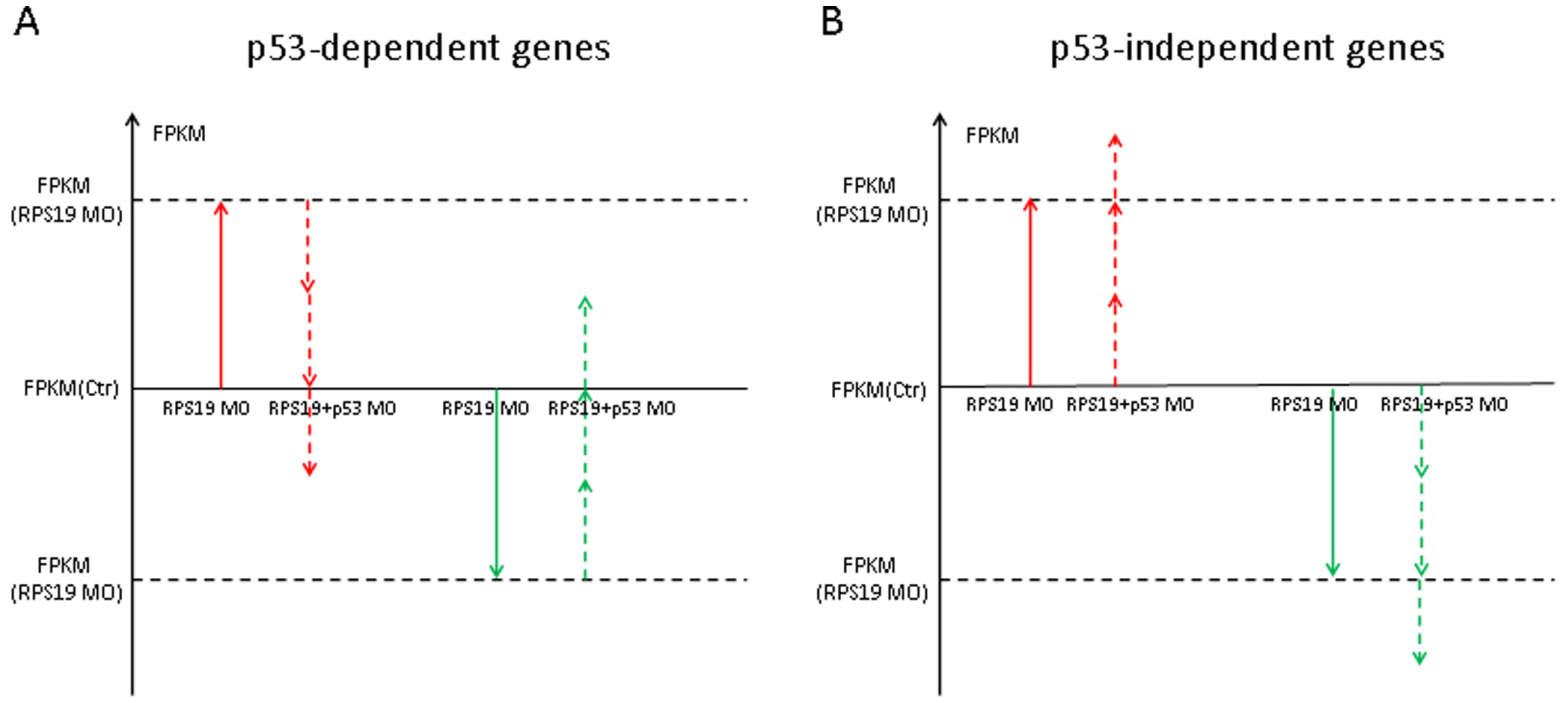
The scheme of detection of p53 dependent and independent genes. The vertical axis represents the gene expression, which is normalized to FPKM. The red arrows indicate the variation trend of FPKM for abnormal up-regulated genes in RPS19 MO, and the green arrows indicate the down-regulated genes in RPS19 MO. Solid arrows indicate the variation trend of FPKM of genes in RPS19 MO, and dashed arrows indicate the variation trend of FPKM of genes in RPS19+p53 MO. Up−/down-regulated genes are screened with the criterion of a fold-change of >2.0 and a p-value of <0.05. (A) Up−/down-regulated genes in RPS19 MO compared with the control and down−/up-regulated genes in RPS19+p53 MO compared with RPS19 MO; we considered genes that satisfied the above criteria to be genes that are p53 dependent. (B) Up−/down-regulated genes in RPS19 MO compared with the control and up−/down-regulated genes in RPS19+p53 MO compared with the control; we considered genes that satisfied the above criteria to be genes that are independent of p53.

Next, we observed the p53-dependent/independent pathways in RPS19-deficient embryos by comparing the transcriptome profiles of control, RPS19 MO and RPS19+p53 MO, using the software Cufflinks. To determine the p53-dependent genes, we firstly searched the up/down-regulated genes in RPS19 MO (compared with control). We then identified genes that their expression levels were reversed (down/up-regulated) in embryos co-injected with RPS19 MO+p53 MO, and defined them as p53 dependent genes. Here we presume that genes whose expression levels did not return to the normal level (control), but did have partial reverse effect compared with pathological state (RPS19 MO), are p53-dependent genes. To determine the p53-independent genes, we presume genes that are up/down-regulated in RPS19 MO (compared with control), and up/down-regulated in RPS19+p53 MO (compared with control), are p53-independent genes. No matter the degree of changes in these genes in RPS19+p53 MO reach the level of them in RPS19 MO or not, as long as the change trends are the same, we accept them as p53-independent genes. (Fig. 7).

Based on the above strategy, we observed 12 potential p53-dependent genes, in which 1 gene was up-regulated and 11 genes were down-regulated (Table 1). The biological functions enriched by these genes are protein folding, integral to membrane and ATP binding. We also found 110 potential p53-independent genes, of which 21 genes were up-regulated and 89 genes were down-regulated. The up-regulated genes are mainly associated with extracellular regions, structural molecule activity, response to stress and intracellular. The down-regulated genes are mainly associated with negative regulation of endopeptidase activity, negative regulation of peptidase activity, and negative regulation of hydrolase activity (p-Value<0.01, number of gene products>2) (Table 2). There were 20 of the regulated pathways that were determined to be p53-dependent (Table S5 in Tables S1) and 76 that were determined to be p53-independent (Table S6 in Tables S1). Real-time PCR and *in situ* hybridization results are mostly consistent with the above sequencing results except for mt2. The real-time PCR results of mt2, fzd5, hsp70l, fn1b, optc, klf11a and srf1 are shown in Fig S2. Interestingly, the mt2 expression in sequencing results is different from the real-time PCR results. We postulated that the discrepancy may arise from: a) the design and optimization of gene-specific real-time primer pairs for mt2; b) the PCR amplification steps bear inherent biases; c) the associated tools for computational analysis are in their infancy, thus may contain errors. It is crucial to identify all existing isoforms in the long run for accurate quantification of a transcriptome. Notably, most of the genes we tested are consistent with the initial RNA-seq experiment data and thus we are confident about the quality of our data. The *in situ* hybridization data of fzd5, alas2, nrp1a and tagln are shown in Fig S3.

**Table 1:** List of p53-dependent genes.

Gene ID	Regulation	Annotation
mt2	up	metallothionein 2
dnajb1b	down	DnaJ (Hsp40) homolog, subfamily B, member 1b
fzd5	down	frizzled homolog 5
hsp70	down	NA
hsp70l	down	heat shock cognate 70-kd protein, like
hsp90aa1.2	down	heat shock protein 90, alpha (cytosolic), class A member 1, tandem duplicate 2
LOC562935	down	NA
mal	down	mal, T-cell differentiation protein
pdcd4b	down	NA
si:dkeyp-35b8.5	down	NA
zgc:136410	down	zgc:136410
zgc:174006	down	zgc:174006

**Table 2:** List of p53-independent genes.

Gene ID	Regulation	Annotation
c6	up	complement component 6
cldn1	up	claudin 1
cyp24a1	up	cytochrome P450, family 24, subfamily A, polypeptide 1
cyr61l1	up	cysteine-rich, angiogenic inducer 61 like 1
fn1b	up	fibronectin 1b
hspb11	up	heat shock protein, alpha-crystallin-related, b11
hspb9	up	heat shock protein, alpha-crystallin-related, 9
LOC554386	up	NA
mustn1	up	NA
optc	up	opticin
pdlim3b	up	PDZ and LIM domain 3b
popdc1	up	popeye domain containing 1
pth1a	up	parathyroid hormone 1a
rn7sk	up	NA
scpp5	up	NA
srfl	up	serum response factor like
tagln	up	transgelin
ydjc	up	NA
zgc:158463	up	zgc:158463
zgc:194878	up	zgc:194878
zgc:92480	up	zgc:92480
a2ml	down	alpha-2-macroglobulin-like
acsl5	down	acyl-CoA synthetase long-chain family member 5
akr1a1a	down	aldo-keto reductase family 1, member A1a (aldehyde reductase)
ambpl	down	alpha-1-microglobulin/bikunin precursor, like
ampd1	down	adenosine monophosphate deaminase 1 (isoform M)
apom	down	NA
barx1	down	BarH-like homeobox 1
bhlhe23	down	basic helix-loop-helix family, member e23
calrl2	down	calreticulin, like 2
cfh	down	complement factor H
col10a1	down	collagen, type X, alpha 1
crx	down	cone-rod homeobox
cyp2aa3v1	down	NA
cyp4v8	down	cytochrome P450, family 4, subfamily V, polypeptide 8
elavl4	down	ELAV (embryonic lethal, abnormal vision, Drosophila)-like 4 (Hu antigen D)
f7	down	coagulation factor VII
f7i	down	coagulation factor VIIi
fabp10a	down	fatty acid binding protein 10a, liver basic
gnb3a	down	NA
habp2	down	hyaluronan binding protein 2
hmga1b	down	high mobility group AT-hook 1b
hmp19	down	HMP19 protein
hpda	down	4-hydroxyphenylpyruvate dioxygenase a
klf11a	down	Kruppel-like factor 11a
krml2	down	NA
lhx9	down	LIM homeobox 9
lipf	down	lipase, gastric
lmo3	down	LIM domain only 3
LOC561143	down	NA
LOC796447	down	NA
mab21l1	down	mab-21-like 1
matn1	down	matrilin 1
msrb2	down	methionine sulfoxide reductase B2
ndrg1b	down	NA
neurod	down	neurogenic differentiation
neurod6a	down	neurogenic differentiation 6a
neurod6b	down	neurogenic differentiation 6b
nfixb	down	nuclear factor I/Xb
nr0b2a	down	nuclear receptor subfamily 0, group B, member 2a
nr2e3	down	nuclear receptor subfamily 2, group E, member 3
otx2	down	orthodenticle homolog 2
otx5	down	orthodenticle homolog 5
oxct1b	down	3-oxoacid CoA transferase 1b
pbx3b	down	pre-B-cell leukemia transcription factor 3b
pcdh1g22	down	protocadherin 1 gamma 22
pcdh1g26	down	protocadherin 1 gamma 26
pcdh8	down	protocadherin 8
pde6g	down	phosphodiesterase 6G, cGMP-specific, rod, gamma
pmepa1	down	prostate transmembrane protein, androgen induced 1
pygl	down	phosphorylase, glycogen; liver (Hers disease, glycogen storage disease type VI)
rbp2a	down	retinol binding protein 2a, cellular
rgs5a	down	regulator of G-protein signaling 5a
samd7	down	NA
sardh	down	sarcosine dehydrogenase
sema3d	down	semaphorin 3d
sept4a	down	septin 4a
serpinc1	down	serine (or cysteine) proteinase inhibitor, clade C (antithrombin), member 1
serpinf1	down	serine (or cysteine) peptidase inhibitor, clade F, member 1
sfrp2	down	secreted frizzled-related protein 2
sh3bgrl2	down	SH3 domain binding glutamic acid-rich protein like 2
si:ch211-102c2.6	down	NA
si:ch211-284e20.8	down	si:ch211-284e20.8
si:dkey-22i16.3	down	NA
si:dkey-39n1.2	down	NA
si:dkey-52k20.7	down	si:dkey-52k20.7
si:dkeyp-86e4.1	down	NA
six7	down	sine oculis homeobox homolog 7
si:xx-by187g17.1	down	NA
slc13a2	down	solute carrier family 13 (sodium-dependent dicarboxylate transporter), member 2
slc17a5	down	solute carrier family 17 (anion/sugar transporter), member 5
slc25a36a	down	solute carrier family 25, member 36a
spon2b	down	spondin 2b, extracellular matrix protein
tfap2b	down	transcription factor AP-2 beta
tuba2	down	tubulin, alpha 2
uox	down	urate oxidase
vasa	down	vasa homolog
vgll4	down	vestigial like 4 (Drosophila)
vmhc	down	ventricular myosin heavy chain
vsx1	down	visual system homeobox 1 homolog, chx10-like
vtna	down	vitronectin a
vtnb	down	vitronectin b
zfpm2b	down	zinc finger protein, multitype 2b
zgc:103710	down	zgc:103710
zgc:158288	down	NA
zgc:158291	down	zgc:158291
zgc:162825	down	zgc:162825
zgc:194131	down	zgc:194131
zgc:77439	down	zgc:77439
zgc:92411	down	zgc:92411

## Discussion

### An Overview of Our Work

In this study, we provided a transcriptome profile of the zebrafish model of DBA, in which the DBA gene RPS19 is knocked-down by MO, for genome-wide analysis using the RNA-Seq technique. We determined differentially expressed genes with statistical significance and enriched pathways and networks by these genes among RPS19 MO, RPS19+p53 MO and control embryos, and we identified p53-dependent and p53-independent genes and pathways by comparing each pair of the three samples. Our data illustrate that not only p53 is the key factor contributing to the significant abnormalities of RPS19-deficient embryos but also some other important factors and pathways participate in regulating the abnormal phenotypes of RPS19-deficient embryos.

### The Reliability of RNA-Seq Data

These samples were sequenced using the Illumina Hi-Seq 2000 Genome Analyzer platform with paired-end 100 base-pair tags to a depth of 35–60 million reads, which is sufficient sequence coverage for transcriptome profiling [14]. We mapped these reads to the zebrafish genome assembly version 2010 (Zv9) using TopHat. Approximately 17–25 million reads could be mapped to the genome, which represents 40%–46% of all of the generated reads. Thus, we believe that the whole genome RNA-Seq data of RPS19-deficient zebrafish transcriptome is of high quality and the genome-wide effects of p53 in RPS19-deficient embryos is reasonable and of scientific significance. It should be noted that the sorted erythrocytes could be a better source for RNA-seq sinc DBA is a red cell disease. However, we could not have enough amount of RNA from blood cells for RNA-seq at the time of experiment. It will be interesting to revisit this when single cell RNA-seq technology is available that are currently being developed.

### The Effects of RPS19 MO and RPS19+p53 MO on Zebrafish Embryos

Zebrafish response to RPS19 deficiency has many features resembling DBA, including impaired ribosome biogenesis, increased apoptosis, developmental abnormalities, and defective hematopoiesis [8]. With the results of the mapping, we finished the gene annotation and differential expression analysis using Cufflinks. For a comparison of the transcriptome of RPS19 MO and control embryos, we observed that the number of down-regulated genes is much more than that of the up-regulated genes in RPS19 MO. This result suggests that in RPS19-deficient embryos, more genes have been inhibited and cannot function properly. IPA provides us more information about gene ontology clusters, enriched pathways and networks of these differentially expressed genes. We found that these genes are mainly associated with cell death, developmental disorder, skeletal and muscular disorders, hematological system development and function, nervous system development and function, and tissue morphology. These results are in agreement with previous studies on ribosomal protein defects and the phenotype of RPS19-deficient zebrafish in this study [8], [15]. Overall, our results indicate that, as a ubiquitous protein, ribosomal protein RPS19 deficiency has very strong effects on zebrafish, and many core factors and pathways are involved in the abnormalities.

The p53 pathway is a key pathway in ribosomal protein defect embryos, with additional roles in regulating other signaling molecules. p53 is always transcriptionally up-regulated when ribosomal protein suffers haplo-insufficiency [5], [6], [16], [17]. However, it is still unclear why the mutations in the ubiquitously expressed RPS19 gene specifically affect erythropoiesis, and whether the transcriptome could return to its original state after p53 knockdown in RPS19-deficient embryos. To clarify the influence of the co-inhibition of p53 in RPS19-deficient embryos, we used RPS19 control Mo, Rps19 Mo, and Rps19 Mo+P53 Mo three groups for the comparative studies. We deduced the effects of p53 MO alone by comparing Rps19 Mo+P53 Mo with Rps19 Mo since we used the same dosage of Rps19 Mo. Our O-staining results showed that the phenotype of Rps19 Mo we obtained in the study can be rescued by the P53 and RPS19 double knockdown, confirming the effectiveness of the P53 Mo knockdown. This indicates that our current study and analysis method do not affect the robustness of the conclusions we made. We observed from the heatmap, which is the clustering of the transcriptome of the three samples, that there is a large difference among the profiles of these samples. Compared with RPS19 MO, the distance between RPS19+p53 MO and the control is smaller, suggesting that, after p53 knockdown, the abnormalities of RPS19-deficient embryos have not been retrieved completely but instead have been alleviated partially. Additionally, the number of differentially expressed genes for RPS19+p53 versus the control is much smaller, and the down-regulated genes are still the majority. IPA analysis demonstrated that the differentially expressed genes are mainly associated with functions of hematological system development and function, cell cycle, cellular development, cellular growth and proliferation, lipid metabolism, molecular transport, and small molecule biochemistry. These results suggest that these functions, regulated by some other important factors, could not rescued by the inhibition of p53 in RPS19-deficienct embryos.

### Less Alleviation Effect of p53 on Hematological Defects

To further study the alleviation effects of p53 on hematological defects caused by RPS19 deficiency, we screened all the genes that are associated with hematopoiesis in the database (The Gene Ontology), and performed matching analysis. We found that, in the differentially expressed genes between RPS19 MO and RPS19+p53 MO, only one gene associated with hematopoiesis was up-regulated in RPS19+p53 MO (fold-change>2.0, p-value<0.05). We supposed that this gene was alleviated by p53 MO.While in RPS19-deficient embryos there are more abnormally down-regulated genes, that are not rescued by p53 MO (Table S4 in Tables S1).This observation indicates that p53 knockdown could not completely rescue the developmental and functional defects of the hematological system in RPS19-deficiency zebrafish. This is expected since hematopoiesis is governed by multiple genes and some of them are not fully controlled by p53. Both our hemoglobin staining observation and RNA-Seq data analysis support the notion that the hematopoiesis is not fully functional in RPS19+p53 MO.

### p53-dependent and -independent Genes and Pathways

In this study we developed a strategy to determine the p53-dependent and -independent genes and pathways from the information on the transcriptome using RNA-Seq data of RPS19 MO, RPS19+p53 MO and the control. Our data shows that the number of p53-independent genes and pathways is significantly more than the number of p53-dependent genes and pathways. This result is in agreement with our hypothesis that many genes and pathways are outside the control of p53. The up-regulated genes link to the functions of structural molecule activity, the extracellular region, response to stress, the intracellular region and more. At the same time, down-regulated genes are associated with the regulation of metabolic processes, the regulation of transcription, sequence-specific DNA binding transcription factor activity and more.

### Conclusions

In conclusion, our study provides an outline of the transcript changes in the zebrafish model of DBA. We can conclude from this study that DBA is a systemic and complex disease due to ribosomal protein defects. DBA causes certain downstream phenotypes, including dysfunction of basic biological and physiological process such as transcription, translation, cellular metabolism, and pathways and networks related to many other disorders. The p53 network is one of the primary pathways in DBA disease. Meanwhile, there are many other p53-independent factors and pathways playing important roles in ribosomal protein defect. Our work has laid foundation in zebrafish DBA model and it will be interesting to see the clinical relevance of zebrafish DBA model in comparison with human DBA data.

## Materials and Methods

### Zebrafish Care

Breeding wild-type zebrafish (Danio rerio) (AB type) were maintained, and embryos were raised under standard library conditions [18]. Zebrafish embryos were kept in a 28.5°C incubator, and the stages (hours post-fertilization) in this study were as described [19]. All of the studies using zebrafish were approved by the Animal Care and Use Committee of Huazhong University of science and technology.

### Microinjection of Morpholino RNA

The rps19 Morpholino (5-CACTGTTACACCACCTGGCATCTTG-3), P53 Morpholino (5- GCGCCATTGCTTTGCAAGAATTG-3), and RPS19 control MO (5-CACTcTTAgACgCACCTGcCATgTTG-3) were obtained from Gene-Tools, LLC. Zebrafish embryos at the one-cell stage were injected with the MOs using a Microinjector (WPI SYS-PV830). Based on the literature and our initial injection trials, 2 ng MO and control Mo was chosen as the optimal concentration. Injected embryos were grown at 28.5°C and were observed under a microscope. The effectiveness of translation inhibition by RPS19 MO (2 ng/embryo) was used by examining *in vivo* the rps19:egfp green fluorescent fusion protein under fluorescence microscopy. We found that at this dosage of 2 ng/embryo, most of the GFP expression was knocked-down.

### Hemoglobin Staining

Hemoglobin in zebrafish embryos was analyzed using o-dianisidine (Sigma) as described [20]. Low-power images were collected using an Olympus microscope with a digital camera (OLYMPUS IX71). Images were imported into Adobe Photoshop CS2 9.0.2 for orientation and figure preparation. O-dianisidine staining results confirmed the effectiveness of the morpholino knockdown while the embryo morphology looks normal.

### RNA Preparation

Immediately after harvesting, 40∼50 pooled embryos at 48 hpf from different experiment replicates were snap-frozen in liquid nitrogen and stored at −80°C. Total RNA was extracted from the pooled embryos using TRIzol (Invitrogen) according to the manufacturer’s instructions. RNA concentrations were determined using NanoDrop 2000 (Thermo Scientific). The integrity of RNA samples was determined using 1.2% Agarose gel electrophoresis, followed by removal of the residual genomic DNA with RNase-free DNaseI (Ambion).

### Library Preparation and Sequencing

mRNA libraries were constructed using the Illumina mRNA-Seq library preparation kit according to the manufacturer’s instructions. The concentration and size distribution of the libraries were determined on an Agilent Bioanalyzer DNA 2000 chip (Agilent Technologies) followed by sequencing on the Illumina Hiseq 2000 Genome Analyser platform in pair-end mode by a 100 bp length. A total of 35–60 million reads were collected for further analysis.

### RNA-Seq Data Analysis

Reads were processed and aligned to the UCSC zebrafish reference genome (build Zv9/danRer7, Jul. 2010) using TopHat (version 1.3.3) [21]. TopHat incorporates the Bowtie v0.12.7 algorithm to perform the alignment. Briefly, TopHat initially removes a portion of reads based on quality information accompanying each read, and maps the qualified reads to the reference genome. The reference index was built using Bowtie with a fasta file for the whole genome of zebrafish downloaded from UCSC (http://genome.ucsc.edu/). The parameters were set by default, but the number of threads to align the reads was set to 6. The aligned read files processed by TopHat were used by Cufflinks (version 1.2.1) software for further analysis, including assembling transcripts, estimating their abundances, and testing for differential expression and regulation in RNA-Seq samples [22]. To calculate gene expression intensity, the read counts were normalized to fragments per kilobase of transcript per million mapped reads (FPKM) according to the gene length and the total mapped reads [23]. Confidence intervals for FPKM estimates were calculated using a Bayesian inference method [24]. Once all of the short read sequences were assembled with Cufflinks, the output.GTF files were sent to Cuffcompare along with a reference.GTF annotation file downloaded from UCSC. This process classified each transcript as known or novel. Cuffcompare produces a combined.GTF file that is passed to Cuffdiff along with the original alignment (.BAM) files produced by TopHat. Cuffdiff then re-estimates the abundance of transcripts listed in the.GTF file using alignments from the.BAM file, and concurrently tests for differential expression. The expression testing is performed at the level of transcripts, primary transcripts and genes [25].

### Cluster Analysis

Average linkage hierarchical clustering of gene expression intensity was performed in this study. The distance (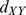) between genes and samples was measured by the Pearson correlation coefficient (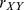), which is 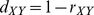. The Pearson correlation coefficient is calculated through
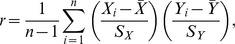
where 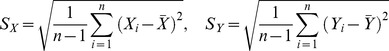 and n is the count of samples, *X_i_* and *Y_i_* are observed values of the two variables, and 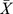 and 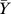 are mean values of the two variables. Computation and visualization were achieved using the heatmap plus package in R. Genes with FPKM greater than 1 in at least one of the 6 samples were chosen to perform cluster analysis.

### IPA Analysis

Gene interaction networks and signal pathways were generated using Ingenuity Pathway Analysis software (http://www.ingenuity.com/). IPA is biological data analysis software from Ingenuity® Systems; it analyzes data from a variety of experimental platforms and provides accurate biological insight into the interactions between genes, proteins, chemicals, pathways, cellular phenotypes, and disease processes.

The differentially expressed genes processed by Cuffdiff and screened with our criteria (fold change>2, p-Value<0.05, we set the p-value to 0.05, which means that the error of discovery of our differential expression genes is 0.05 for statistical significance. It is a meaningful measurement similar to FDR and routinely used in such studies.) Genes identified to be significant were then submitted to IPA for biological function, canonical pathway, and interaction network analysis. Because the current edition of IPA cannot accept and identify the gene ID of zebrafish yet, we must match these differentially expressed genes of zebrafish to their homologs of human genes. The homolog genes database that we used was HomoloGene, which was downloaded from NCBI. After converting to human homolog genes, we submitted these homologs to IPA. IPA identified a set of genes that are enriched for a specific function or pathway, and the enrichment is represented as a ratio. To find the significance of the enrichment in a specific function, IPA calculates the significance value based on the measure of involvement of the gene in the input data set to their respective molecular function/signaling pathways [26]. The significance of the networks is calculated using Fisher’s exact test, and the p-value is the executed negative logarithmic transformation.

### Quantitative PCR (qPCR)

2 µg total RNA was used for reverse transcription with TransScript First-strand cDNA Synthesis SuperMix (TransGen). cDNA was then diluted 1∶5 with RNase free H_2_O and 2 µL of this cDNA was used in the qPCR reaction. Quantitative PCR (qPCR) was performed using GoTaq® qPCR Master Mix (Promega) on an Applied Biosystems 7900 Real-Time PCR System (ABI 7900). The data were normalized against zebrafish β-actin. The sequences of the primers used for qPCR are given in Table S7 in Tables S1.

### Whole Mount in situ Hybridization

For in situ hybridization, the following genes were used as probes: nrp1a, alas2, tagln and fzd5. cDNAs were amplified by RT-PCR and the products were cloned into pTA2 vector (TOYOBO). The sequences of the primers used for probe are given in Table S8 in Tables S1. Antisense probes were synthesized using T7 RNA polymerases (Promega) and labeled with DIG RNA labeling Mix (Roche). Embryos at 48 hpf were fixed in 4% PFA/PBS overnight at 4°C, dehydrated in 100% methanol and stored at −20°C before use. Rehydrated embryos were prehybridized for 3 h at 65°C followed by overnight hybridization with DIG-labeled probes. After washing, embryos were incubated with 2% blocking solution, followed by incubation with anti-Digoxlgenin-Ap (Roche), 1∶4000 in blocking solution. After extensive washing, samples are visualized by incubating with NBT/BCIP AP stock solution (Roche) in AP buffer. Photographs were taken by using a stereo fluorescence microscope (OLYMPUS SZX2-ILLB).

### Data Access

The raw sequence data can be accessed from the Gene Expression Omnibus, accession number is GSE45699 (available upon request or online when paper get accepted), and all of the genomic resources generated in this study are provided as Supplementary Files.

## Supporting Information

Figure S1 **RPS19 is not required for HSC formation and RPS19 knockdown embryos have normal number of gata1-positive cells.** (A–C) The expression of cmyb (black arrow) was comparable in RPS19 morphants and RPS19 and P53 double morphants at 48 hpf. (D–F) The expression of runx1 was comparable in RPS19 morphants and RPS19 and P53 double morphants at 48 hpf. (G–I) The expression of gata1 was comparable in RPS19 morphants and RPS19 and P53 double morphants at 24 hpf. A–I is lateral view.
(JPG)

Figure S2 **Real-time PCR results of the mt2, fzd5, hsp70l, klf11a, srf1 and fn1b.**
(TIF)

Figure S3 **The in situ results of fzd5, alas2, nrp1a and tagln in zebrafish embryos at 48hpf.** (A–C) The expression of fzd5 (black arrow) in control, RPS19 morphants and RPS19 and P53 double morphants at 48 hpf. (D–F) The expression of alas2 (black arrow) in control, RPS19 morphants and RPS19 and P53 double morphants at 48 hpf. (G–I) The expression of nrp1a (black arrow) in control, RPS19 morphants and RPS19 and P53 double morphants at 48 hpf. (J–L) The expression of tagln (black arrow) in control, RPS19 morphants and RPS19 and P53 double morphants at 48 hpf. A-L is lateral view.
(TIF)

Tables S1 **File includes Tables S1-S8.**
(DOC)

## References

[1] Gazda HT, Sieff CA (2006) Recent insights into the pathogenesis of Diamond-Blackfan anaemia. Br J Haematol 135: 149–157. 16942586 10.1111/j.1365-2141.2006.06268.x

[2] Dianzani I, Loreni F (2008) Diamond-Blackfan anemia: a ribosomal puzzle. Haematologica 93: 1601–1604. 18978295 10.3324/haematol.2008.000513

[3] Duan J, Ba Q, Wang Z, Hao M, Li X, et al (2011) Knockdown of ribosomal protein S7 causes developmental abnormalities via p53 dependent and independent pathways in zebrafish. Int J Biochem Cell Biol 43: 1218–1227. 21550419 10.1016/j.biocel.2011.04.015

[4] Gomez G, Lee JH, Veldman MB, Lu J, Xiao X, et al (2012) Identification of vascular and hematopoietic genes downstream of etsrp by deep sequencing in zebrafish. PLoS One 7: e31658. 22438865 10.1371/journal.pone.0031658 PMC3306315

[5] Taylor AM, Humphries JM, White RM, Murphey RD, Burns CE, et al.. (2012) Hematopoietic defects in rps29 mutant zebrafish depend upon p53 activation. Exp Hematol 40: 228–237 e225. 10.1016/j.exphem.2011.11.007 PMC3310385 22120640

[6] Danilova N, Sakamoto KM, Lin S (2011) Ribosomal protein L11 mutation in zebrafish leads to haematopoietic and metabolic defects. Br J Haematol 152: 217–228. 21114664 10.1111/j.1365-2141.2010.08396.x PMC3457809

[7] Torihara H, Uechi T, Chakraborty A, Shinya M, Sakai N, et al (2011) Erythropoiesis failure due to RPS19 deficiency is independent of an activated Tp53 response in a zebrafish model of Diamond-Blackfan anaemia. Br J Haematol 152: 648–654. 21223253 10.1111/j.1365-2141.2010.08535.x

[8] Danilova N, Sakamoto KM, Lin S (2008) Ribosomal protein S19 deficiency in zebrafish leads to developmental abnormalities and defective erythropoiesis through activation of p53 protein family. Blood 112: 5228–5237. 18515656 10.1182/blood-2008-01-132290

[9] Zhang Y, Duc ACE, Rao SY, Sun XL, Bilbee AN, et al (2013) Control of Hematopoietic Stem Cell Emergence by Antagonistic Functions of Ribosomal Protein Para logs. Developmental Cell 24: 411–425. 23449473 10.1016/j.devcel.2013.01.018 PMC3586312

[10] Poulain M, Ober EA (2011) Interplay between Wnt2 and Wnt2bb controls multiple steps of early foregut-derived organ development. Development 138: 3557–3568. 21771809 10.1242/dev.055921 PMC3143568

[11] Burns CJ, Zhang JM, Brown EC, Van Bibber AM, Van Es J, et al (2008) Investigation of Frizzled-5 during embryonic neural development in mouse. Developmental Dynamics 237: 1614–1626. 18489003 10.1002/dvdy.21565 PMC2562763

[12] Mendt M, Cardier JE (2012) Stromal-Derived Factor-1 and Its Receptor, CXCR4, Are Constitutively Expressed by Mouse Liver Sinusoidal Endothelial Cells: Implications for the Regulation of Hematopoietic Cell Migration to the Liver During Extramedullary Hematopoiesis. Stem Cells and Development 21: 2142–2151. 22121892 10.1089/scd.2011.0565 PMC3411357

[13] Luis TC, Ghazvini M, Naber BAE, de Haas EFE, van Dongen JJM, et al (2010) Canonical Wnt Signaling Regulates Hematopoiesis in a Dosage-Dependent Fashion. Experimental Hematology 38: S95–S95. 10.1016/j.stem.2011.07.017 21982234

[14] Sultan M, Schulz MH, Richard H, Magen A, Klingenhoff A, et al (2008) A global view of gene activity and alternative splicing by deep sequencing of the human transcriptome. Science 321: 956–960. 18599741 10.1126/science.1160342

[15] Uechi T, Nakajima Y, Chakraborty A, Torihara H, Higa S, et al (2008) Deficiency of ribosomal protein S19 during early embryogenesis leads to reduction of erythrocytes in a zebrafish model of Diamond-Blackfan anemia. Hum Mol Genet 17: 3204–3211. 18653748 10.1093/hmg/ddn216

[16] Chakraborty A, Uechi T, Higa S, Torihara H, Kenmochi N (2009) Loss of ribosomal protein L11 affects zebrafish embryonic development through a p53-dependent apoptotic response. PLoS One 4: e4152. 19129914 10.1371/journal.pone.0004152 PMC2612748

[17] Dutt S, Narla A, Lin K, Mullally A, Abayasekara N, et al (2011) Haploinsufficiency for ribosomal protein genes causes selective activation of p53 in human erythroid progenitor cells. Blood 117: 2567–2576. 21068437 10.1182/blood-2010-07-295238 PMC3062351

[18] Westerfield M, Doerry E, Douglas S (1999) Zebrafish in the Net. Trends Genet 15: 248–249. 10354586 10.1016/s0168-9525(99)01741-2

[19] Kimmel CB, Ballard WW, Kimmel SR, Ullmann B, Schilling TF (1995) Stages of embryonic development of the zebrafish. Dev Dyn 203: 253–310. 8589427 10.1002/aja.1002030302

[20] Detrich HW (1995) Intraembryonic hematopoietic cell migration during vertebrate development. Proc Natl Acad Sci U S A 92: 10713–10717. 7479870 10.1073/pnas.92.23.10713 PMC40682

[21] Trapnell C, Williams BA, Pertea G, Mortazavi A, Kwan G, et al (2010) Transcript assembly and quantification by RNA-Seq reveals unannotated transcripts and isoform switching during cell differentiation. Nat Biotechnol 28: 511–515. 20436464 10.1038/nbt.1621 PMC3146043

[22] Roberts A, Trapnell C, Donaghey J, Rinn JL, Pachter L (2011) Improving RNA-Seq expression estimates by correcting for fragment bias. Genome Biol 12: R22. 21410973 10.1186/gb-2011-12-3-r22 PMC3129672

[23] Trapnell C, Williams BA, Pertea G, Mortazavi A, Kwan G, et al (2010) Transcript assembly and quantification by RNA-Seq reveals unannotated transcripts and isoform switching during cell differentiation. Nature Biotechnology 28: 511–U174. 10.1038/nbt.1621 PMC3146043 20436464

[24] Jiang H, Wong WH (2009) Statistical inferences for isoform expression in RNA-Seq. Bioinformatics 25: 1026–1032. 19244387 10.1093/bioinformatics/btp113 PMC2666817

[25] Twine NA, Janitz K, Wilkins MR, Janitz M (2011) Whole Transcriptome Sequencing Reveals Gene Expression and Splicing Differences in Brain Regions Affected by Alzheimer’s Disease. PLoS One 6. 10.1371/journal.pone.0016266 PMC3025006 21283692

[26] Hegde A, Qiu NC, Qiu XH, Ho SHK, Tay KQY, et al.. (2008) Genomewide Expression Analysis in Zebrafish mind bomb Alleles with Pancreas Defects of Different Severity Identifies Putative Notch Responsive Genes. PLoS One 3. 10.1371/journal.pone.0001479 PMC2195453 18213387

