# Correction: Transcriptome Analysis of the Zebrafish Model of Diamond-Blackfan Anemia from RPS19 Deficiency via p53-Dependent and -Independent Pathways

**DOI:** 10.1371/annotation/54600aa1-0df0-4e0a-b2ab-9d6cdd46cade

**Published:** 2013-08-22

**Authors:** Qiong Jia, Qian Zhang, Zhaojun Zhang, Yaqin Wang, Wanguang Zhang, Yang Zhou, Yang Wan, Tao Cheng, Xiaofan Zhu, Xiangdong Fang, Weiping Yuan, Haibo Jia

There was an error in the Funding statement. The correct statement is:

This work was supported by the following funds from the Ministry of Science and Technology (2013CB945300, 2012CB966600, 2011AA020118, 2011CB964800); NSFC (81090410, 81130074, 31171387, 31000640, 81170465); the National Key Scientific Instrument 23/35 and Equipment Development Projects of China (2011YQ03013404); the “Strategic Priority Research Program” of the Chinese Academy of Sciences, Stem Cell and Regenerative Medicine Research (XDA01040405); Hubei National Natural Science Foundation Grant 2010CDB02402 and State Key Laboratory of Experimental Hematology Pilot Project Grant ZK12-05. The funders had no role in study design, data collection and analysis, decision to publish, or preparation of the manuscript. 

