# Correction: Transcriptome Analysis of the Zebrafish Model of Diamond-Blackfan Anemia from RPS19 Deficiency via p53-Dependent and -Independent Pathways

**DOI:** 10.1371/annotation/df71e418-b886-49ea-bb6a-b96341b27cfc

**Published:** 2013-11-11

**Authors:** Qiong Jia, Qian Zhang, Zhaojun Zhang, Yaqin Wang, Wanguang Zhang, Yang Zhou, Yang Wan, Tao Cheng, Xiaofan Zhu, Xiangdong Fang, Weiping Yuan, Haibo Jia

In the author byline, the first two authors, Qiong Jia and Qian Zhang, should be noted as equal contributors.

The last two authors, Weiping Yuan and Haibo Jia, should be noted as the corresponding authors. The emails addresses are, respectively, wpyuan@ihcams.ac.cn and haibo.jia@mail.hust.edu.cn

