# Supplementary figures and images for: Transcriptome Analysis of the Zebrafish Model of Diamond-Blackfan Anemia from RPS19 Deficiency via p53-Dependent and -Independent Pathways
Source: PLoS One. 2013 Aug 19;8(8):e71782. doi: 10.1371/journal.pone.0071782 (PMC3747179; doi:10.1371/journal.pone.0071782)

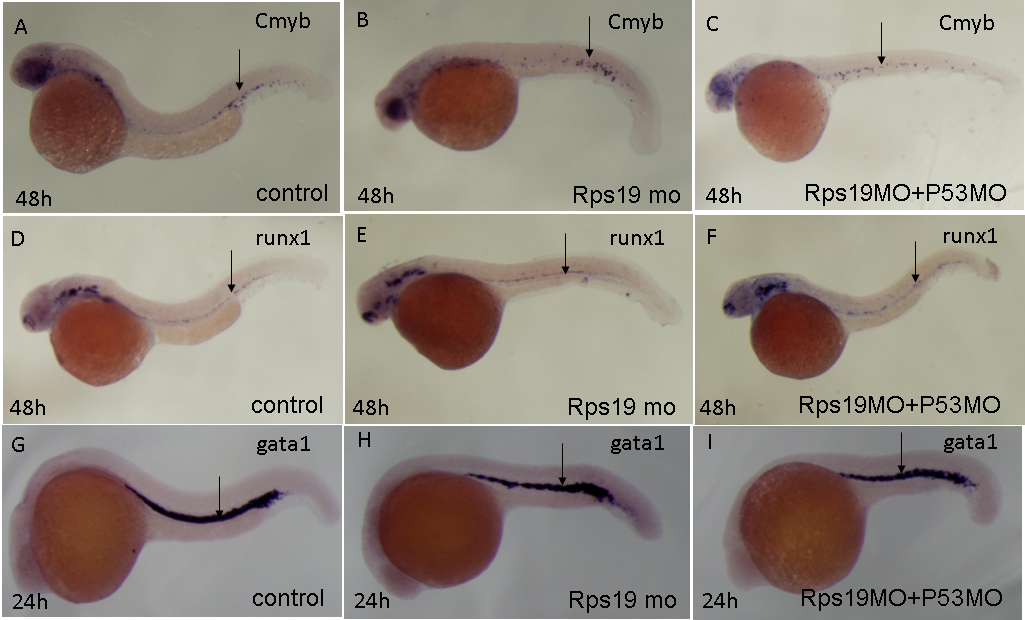

Supplement: Figure S1 — RPS19 is not required for HSC formation and RPS19 knockdown embryos have normal number of gata1-positive cells. (A–C) The expression of cmyb (black arrow) was comparable in RPS19 morphants and RPS19 and P53 double morphants at 48 hpf. (D–F) The expression of runx1 was comparable in RPS19 morphants and RPS19 and P53 double morphants at 48 hpf. (G–I) The expression of gata1 was comparable in RPS19 morphants and RPS19 and P53 double morphants at 24 hpf. A–I is lateral view. (JPG) [file pone.0071782.s001.jpg]

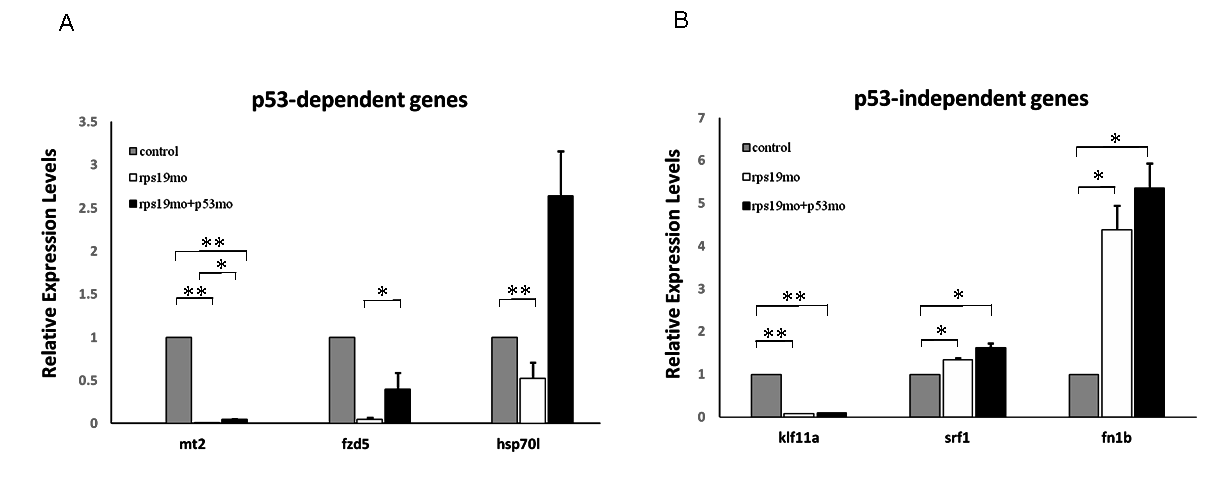

Supplement: Figure S2 — Real-time PCR results of the mt2, fzd5, hsp70l, klf11a, srf1 and fn1b. (TIF) [file pone.0071782.s002.tif]

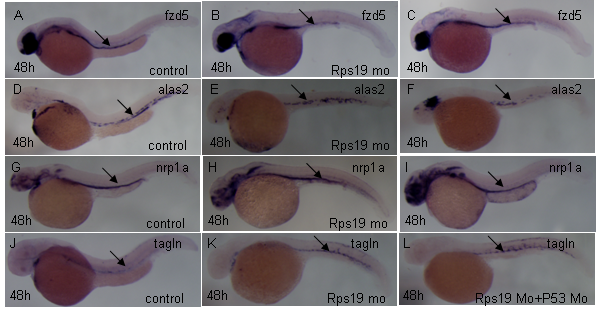

Supplement: Figure S3 — The in situ results of fzd5, alas2, nrp1a and tagln in zebrafish embryos at 48hpf. (A–C) The expression of fzd5 (black arrow) in control, RPS19 morphants and RPS19 and P53 double morphants at 48 hpf. (D–F) The expression of alas2 (black arrow) in control, RPS19 morphants and RPS19 and P53 double morphants at 48 hpf. (G–I) The expression of nrp1a (black arrow) in control, RPS19 morphants and RPS19 and P53 double morphants at 48 hpf. (J–L) The expression of tagln (black arrow) in control, RPS19 morphants and RPS19 and P53 double morphants at 48 hpf. A-L is lateral view. (TIF) [file pone.0071782.s003.tif]
