# Supplementary material for: Transcriptome Analysis of the Zebrafish Model of Diamond-Blackfan Anemia from RPS19 Deficiency via p53-Dependent and -Independent Pathways
Source: PLoS One. 2013 Aug 19;8(8):e71782. doi: 10.1371/journal.pone.0071782 (PMC3747179; doi:10.1371/journal.pone.0071782)
Supplement: Tables S1 — File includes Tables S1-S8. (DOC) [file pone.0071782.s004.doc]

**Table S1 Summary of mapping results**

|  | Control | | RPS19 MO | | RPS19+p53 MO | |
| --- | --- | --- | --- | --- | --- | --- |
|  | Left | Right | Left | Right | Left | Right |
| Total reads | 19,909,797 | 19,909,797 | 29,429,804 | 29,429,804 | 20,378,171 | 20,378,171 |
| Reads filtered out by TopHat | 102,356 | 64,572 | 112,731 | 51,195 | 67,873 | 24,762 |
| Reads processed | 19,807,441 | 19,845,225 | 29,317,073 | 29,378,609 | 20,310,298 | 20,353,409 |
| Total mapped reads | 8,733,521 | 8,313,768 | 12,471,036 | 11,807,556 | 9,422,397 | 9,084,900 |
| Mapping percent | 44.09% | 41.89% | 42.54% | 40.19% | 46.39% | 44.64% |

**Table S2 List of pathways that are enriched by differentially expressed genes in RPS19 MO (p-value<0.05)**

| Ingenuity Canonical Pathways | p-value | Molecules |
| --- | --- | --- |
| Coagulation System | 1.45E-05 | PLG, SERPINC1, F9, A2M, SERPINF2, F2 |
| Acute Phase Response Signaling | 5.63E-05 | PLG, ITIH3, ITIH4, AMBP, SERPINF1, RBP2, CP, A2M, SERPINF2, F2, CRABP1 |
| LXR/RXR Activation | 5.89E-04 | APOE, APOA4, ITIH4, VTN, AMBP, SERPINF1, CLU, SERPINF2 |
| Intrinsic Prothrombin Activation Pathway | 7.41E-04 | SERPINC1, F9, COL10A1, F2 |
| Taurine and Hypotaurine Metabolism | 9.33E-04 | GAD2, GAD1(includes EG:100006588), CSAD |
| Butanoate Metabolism | 1.74E-03 | GAD2, GAD1(includes EG:100006588), OXCT1, DCXR, ELOVL6 |
| Maturity Onset Diabetes of Young (MODY) Signaling | 2.95E-03 | NEUROD1, ALDOB, INS |
| GABA Receptor Signaling | 6.03E-03 | DNM1, GAD2, GAD1(includes EG:100006588), SLC6A1 |
| Phototransduction Pathway | 8.51E-03 | PDE6G, GNB3, RHO, OPN1LW |
| FXR/RXR Activation | 9.77E-03 | APOE, NR0B2, INS, FBP1, VLDLR |
| Pentose Phosphate Pathway | 0.010 | ALDOB, RGN, FBP1 |
| Complement System | 0.013 | C8B, CFH, C8A |
| Aryl Hydrocarbon Receptor Signaling | 0.017 | CCNE2, HSP90B1, NR0B2, NFIA, DHFR, CYP1B1 |
| Alanine and Aspartate Metabolism | 0.018 | GAD2, PC, GAD1(includes EG:100006588) |
| Clathrin-mediated Endocytosis Signaling | 0.023 | DNM1, APOE, APOA4, AMPH, INS, CLU, F2 |
| Extrinsic Prothrombin Activation Pathway | 0.025 | SERPINC1, F2 |
| Glyoxylate and Dicarboxylate Metabolism | 0.028 | HAO2, HOGA1 |
| Fructose and Mannose Metabolism | 0.029 | SORD, ALDOB, FBP1 |
| Endoplasmic Reticulum Stress Pathway | 0.031 | DNAJC3, HSPA5 |
| Histidine Metabolism | 0.031 | FTCD, UROC1, ELOVL6 |
| Aldosterone Signaling in Epithelial Cells | 0.034 | HSP90B1, HSPA1A/HSPA1B, PDIA3, DNAJC3, DNAJB1, HSPA5 |
| One Carbon Pool by Folate | 0.035 | FTCD, DHFR |
| Lipid Antigen Presentation by CD1 | 0.035 | CALR, PDIA3 |
| Wnt/β-catenin Signaling | 0.044 | SOX4, SFRP2, SOX1, FZD5, SFRP1, TCF7L2 |
| Huntington's Disease Signaling | 0.049 | NEUROD1, DNM1, GNB3, HSPA1A/HSPA1B, CAPN2, DNAJB1, HSPA5 |

**Table S3 List of pathways that are enriched by differentially expressed genes in RPS19+p53 MO (p-value<0.05)**

| Ingenuity Canonical Pathways | p-value | Molecules |
| --- | --- | --- |
| LXR/RXR Activation | 3.47E-05 | APOA4, VTN, AMBP, SERPINF1, AGT, CETP |
| Acute Phase Response Signaling | 2.00E-04 | AMBP, SERPINF1, RBP2, SERPINE1, A2M, AGT |
| Coagulation System | 7.41E-04 | SERPINC1, SERPINE1, A2M |
| Glycine, Serine and Threonine Metabolism | 5.50E-03 | SARDH, GNMT, ALAS2 |
| Intrinsic Prothrombin Activation Pathway | 8.32E-03 | SERPINC1, COL10A1 |
| Huntington's Disease Signaling | 0.023 | NEUROD1, TGM2, GNB3, CAPN2 |
| Phototransduction Pathway | 0.029 | PDE6G, GNB3 |
| Aryl Hydrocarbon Receptor Signaling | 0.031 | TGM2, NR0B2, HSPB7 |
| Hepatic Fibrosis / Hepatic Stellate Cell Activation | 0.033 | MYH7, A2M, AGT |

**Table S4 List of the differential expression of hematopoietic genes**

| Category | Gene symbol | log2(fold change) |
| --- | --- | --- |
| Up-regulated in RPS19 MO (vs. Control) | / | / |
| Down-regulated in RPS19 MO (vs. Control) | bmper | -1.57 |
|  | fzd5 | -1.75 |
|  | nrp1a | -1.07 |
|  | sema3d | -2.22 |
|  | tbx1 | -1.73 |
| Up-regulated in RPS19+p53 MO (vs. Control) | / | / |
| Down-regulated in RPS19+p53 MO (vs. Control) | agxt2l1 | -1.52 |
|  | alas2 | -1.83 |
|  | sema3d | -1.94 |
| Up-regulated in RPS19+p53 MO (vs. RPS19 MO) | fzd5 | 2.07 |
| Down-regulated in RPS19+p53 MO (vs. RPS19 MO) | / | / |

**Table S5 List of p53-dependent pathways**

| Pathways | Regulation |
| --- | --- |
| Leukocyte Extravasation Signaling | up |
| Aldosterone Signaling in Epithelial Cells | down |
| Androgen Signaling | down |
| Basal Cell Carcinoma Signaling | down |
| Colorectal Cancer Metastasis Signaling | down |
| DNA Double-Strand Break Repair by Homologous Recombination | down |
| eNOS Signaling | down |
| Factors Promoting Cardiogenesis in Vertebrates | down |
| Glioblastoma Multiforme Signaling | down |
| Glucocorticoid Receptor Signaling | down |
| Human Embryonic Stem Cell Pluripotency | down |
| Huntington's Disease Signaling | down |
| Mouse Embryonic Stem Cell Pluripotency | down |
| NRF2-mediated Oxidative Stress Response | down |
| Ovarian Cancer Signaling | down |
| Protein Ubiquitination Pathway | down |
| Role of Macrophages, Fibroblasts and Endothelial Cells in Rheumatoid Arthritis | down |
| Role of Osteoblasts, Osteoclasts and Chondrocytes in Rheumatoid Arthritis | down |
| Role of Wnt/GSK-3β Signaling in the Pathogenesis of Influenza | down |
| Wnt/β-catenin Signaling | down |

**Table S6 List of p53-independent pathways**

| Pathways | Regulation |
| --- | --- |
| Amyloid Processing | up |
| Amyotrophic Lateral Sclerosis Signaling | up |
| Apoptosis Signaling | up |
| Calcium-induced T Lymphocyte Apoptosis | up |
| ERK/MAPK Signaling | up |
| FAK Signaling | up |
| Glutathione Metabolism | up |
| Huntington's Disease Signaling | up |
| Integrin Signaling | up |
| Leukocyte Extravasation Signaling | up |
| Mitochondrial Dysfunction | up |
| Nicotinate and Nicotinamide Metabolism | up |
| nNOS Signaling in Neurons | up |
| Sertoli Cell-Sertoli Cell Junction Signaling | up |
| Tight Junction Signaling | up |
| VDR/RXR Activation | up |
| Actin Cytoskeleton Signaling | down |
| Acute Phase Response Signaling | down |
| Androgen Signaling | down |
| Antigen Presentation Pathway | down |
| Antiproliferative Role of Somatostatin Receptor 2 | down |
| Aryl Hydrocarbon Receptor Signaling | down |
| Atherosclerosis Signaling | down |
| Breast Cancer Regulation by Stathmin1 | down |
| Butanoate Metabolism | down |
| Calcium Signaling | down |
| Cardiac β-adrenergic Signaling | down |
| Cardiomyocyte Differentiation via BMP Receptors | down |
| CCR5 Signaling in Macrophages | down |
| Cellular Effects of Sildenafil (Viagra) | down |
| Clathrin-mediated Endocytosis Signaling | down |
| Coagulation System | down |
| Colorectal Cancer Metastasis Signaling | down |
| Complement System | down |
| Docosahexaenoic Acid (DHA) Signaling | down |
| Ephrin B Signaling | down |
| Extrinsic Prothrombin Activation Pathway | down |
| Fatty Acid Metabolism | down |
| FXR/RXR Activation | down |
| Glioma Invasiveness Signaling | down |
| Glucocorticoid Receptor Signaling | down |
| Glutamate Metabolism | down |
| Glutamate Receptor Signaling | down |
| Glycine, Serine and Threonine Metabolism | down |
| G Protein Signaling Mediated by Tubby | down |
| Growth Hormone Signaling | down |
| Hepatic Cholestasis | down |
| Hepatic Fibrosis / Hepatic Stellate Cell Activation | down |
| Huntington's Disease Signaling | down |
| IL-12 Signaling and Production in Macrophages | down |
| Intrinsic Prothrombin Activation Pathway | down |
| Lipid Antigen Presentation by CD1 | down |
| LPS/IL-1 Mediated Inhibition of RXR Function | down |
| LXR/RXR Activation | down |
| Maturity Onset Diabetes of Young (MODY) Signaling | down |
| Mitochondrial Dysfunction | down |
| Neuroprotective Role of THOP1 in Alzheimer's Disease | down |
| P2Y Purigenic Receptor Signaling Pathway | down |
| P70S6K Signaling | down |
| Phototransduction Pathway | down |
| PI3K Signaling in B Lymphocytes | down |
| PPARα/RXRα Activation | down |
| PPAR Signaling | down |
| Production of Nitric Oxide and Reactive Oxygen Species in Macrophages | down |
| Protein Kinase A Signaling | down |
| Purine Metabolism | down |
| PXR/RXR Activation | down |
| Relaxin Signaling | down |
| Role of Osteoblasts, Osteoclasts and Chondrocytes in Rheumatoid Arthritis | down |
| Signaling by Rho Family GTPases | down |
| Synthesis and Degradation of Ketone Bodies | down |
| Thrombin Signaling | down |
| Type II Diabetes Mellitus Signaling | down |
| Urea Cycle and Metabolism of Amino Groups | down |
| Valine, Leucine and Isoleucine Degradation | down |
| Wnt/β-catenin Signaling | down |

**Table S7 List of qPCR primers used in this study**

| Gene | Forward sequence | Reverse sequence |
| --- | --- | --- |
| β-actin | 5′-ATTGCTGACAGGATGCAGAAG-3' | 5′-GATGGTCCAGACTCATCGTACTC-3' |
| mt2 | 5′-AGACTGGAACTTGCAACTGTGGT-3′ | 5′-CAGCTGGAGCCACAGGAATT-3′ |
| fzd5 | 5'-GGTGACCAGCAGCACATTCTCT-3' | 5'-CCAAGCCGCAAGGTGGAAGTAT-3' |
| Hsp70l | 5'-TCATCAAGCGCAACACAACC-3' | 5′-GGATTCTGACTGACAGACGATG -3′ |
| bmper | 5'-AACTCACCACCATCGCAGGACT-3' | 5'-TCGCCGCTGACCGTTGTAGT-3' |
| sema3d | 5'-CCACCTCCACACCAGCAGTACA-3' | 5'-GACACGAGACAGCACGCTCTTG-3' |
| tbx1 | 5'-TTACCGCCGTCACAGCCTATCA-3' | 5'-CCAGTCCTCGGGATCACAGTCT-3' |
| cyp24a1 | 5'-GTCGGACATCTTCCATAACG-3' | 5'-GGCACGGACGGTGAAACTC-3' |
| fn1b  optc  klf11a  srf1 | 5’-GACACGACCACACGACACTG-3’  5’-AGCATGAGGAGGTTGAGACAGT-3’  5’-CCACCTCGGACTCCTGTGATTC-3’  5’-GATACACGACCTTCAGCAAGA-3’ | 5’-CAACGCTCCGCAACAGGTT-3’  5’-ACAGCAGGTGGAGGAGTGAG-3’  5’-TGAGAACCGCTGTGCCTGAA-3’  5’-TTCACTGGCAACCAAGAGTAA |
|  |  |  |

**Table S8 List of probe primers used in this study**

| Gene | Forward sequence | Reverse sequence |
| --- | --- | --- |
| nrp1a | 5’-GAGTCTTCTGCCGATACG-3’ | 5’-GTCCACCTTATATTCCTTCAC-3’ |
| alas2 | 5’-GGACCACACCTACCGTAT-3’ | 5’-CTCACAACCTGGAAGCATT-3’ |
| tagln | 5’-GCAGGATAAGATCGAGCAGAAG-3’ | 5’CCTCCTTCATCTGATCGTCTGA-3’ |
| fzd5 | 5’-GGTGACCAGCAGCACATTCTCT-3’ | 5’-CCAAGCCGCAAGGTGGAAGTAT-3‘ |
